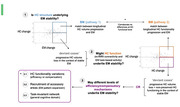# Insights from Functional MRI: Understanding the Mechanisms underlying Reserve and Brain Maintenance

**DOI:** 10.1002/alz.087231

**Published:** 2025-01-09

**Authors:** David Bartrés‐Faz, María Cabello‐Toscano, Micael Andersson, Didac Vidal‐Piñeiro, Alvaro Pascual‐Leone, Kristine B Walhovd, Anders M Fjell, Lars Nyberg, Lídia Vaqué‐Alcázar

**Affiliations:** ^1^ Institute of Biomedical Research August Pi i Sunyer (IDIBAPS), Barcelona Spain; ^2^ Institut Guttmann, Institut Universitari de Neurorehabilitació adscrit a la UAB, Badalona, Barcelona Spain; ^3^ Department of Medicine, Faculty of Medicine and Health Sciences, Institute of Neurosciences, University of Barcelona, Barcelona, Spain. Institut d’Investigacions Biomèdiques August Pi i Sunyer (IDIBAPS), Barcelona Spain; ^4^ Department of Medicine, Faculty of Medicine and Health Sciences, Institute of Neurosciences, University of Barcelona, Barcelona Spain; ^5^ Umeå Center for Functional Brain Imaging, Umeå University, Umeå Sweden; ^6^ LCBC, University of Oslo, Oslo Norway; ^7^ Hinda and Arthur Marcus Institute for Aging Research and Deanna and Sidney Wolk Center for Memory Health, Hebrew SeniorLife, Boston, MA USA; ^8^ Sant Pau Memory Unit, Hospital de la Santa Creu i Sant Pau, Biomedical Research Institute Sant Pau, Universitat Autònoma de Barcelona, Barcelona Spain

## Abstract

**Background:**

Cognitive reserve (CR) and Brain Maintenance (BR) are constructs defined at a theoretical level (Stern et al., Neurobiol Aging, 2021 Apr, 124:100‐103). Our aim was to propose a reproducible procedure to compare CR‐like of BR‐like mechanisms underlying interindividual differences in memory stability.

**Method:**

Leveraging data from the Lifebrain consortia (Walhovd et al. Eur Psychiatry. 2018 Jan;47:76‐87) we gathered information regarding 1) episodic memory (EM) stability, defined as those subjects showing no negative memory changes across two time point assessments (i.e., change ≥0), 2) brain structure and 3) brain functionality (resting‐state functional magnetic resonance imaging [MRI]) changes). We designed a unified approach where at each step an analysis between EM and multimodal MRI‐based measures provides a general metric, referring to either associations between EM and hippocampal (HC) volume changes, or to EM and fMRI connectivity changes (see Figure). This aimed to distinguish a ‘BM – Pathway 1’ identifying those subjects presenting a correspondence between both stability of EM and of HC volumes (i.e x≥0 & y≥0 quadrants of the upper left scatterplot) and a, ‘BM ‐ pathway 2’ reflecting a correspondence between stability of EM and HPC fMRI functionality (i.e x≥0 & y≥0 quadrants of the bottom right scatterplot). Finally, a CR ‐ pathway (‘deviant cases’ in Figure) was defined for subjects where a discrepancy between EM stability and their neural substrates was found (i.e. x>0 and y<0 quadrants in both scatterplots).

**Result:**

Findings obtained from N = 532 participants (67.8 years at baseline, 294 women) revealed that N = 275 exhibited memory stability over time. Present analyses show that from those, N = 166 (60%) could be classified as BM ‐pathway 1 at the first step, whereas N = 109 (40%) cases were classified as CR ‐ pathway. Further results from a subsample, indicate that CR ‐ pathway cases exhibited a positive change in EM and negative in HC volumes, evidencing reductions in functional connectivity between HC and the Medial Prefrontal node from the dorsal Default Mode Network.

**Conclusion:**

The present approach combining structural and functional MRI to study CR and BM constructs shall provide new relevant empirical data helping to clarify the conceptual boundaries between these categories.